# The presence of children in households was associated with dietary intake among Japanese married women: the POTATO study

**DOI:** 10.1017/jns.2018.9

**Published:** 2018-04-12

**Authors:** Aki Saito, Mai Matsumoto, Aiko Hyakutake, Masafumi Saito, Naoko Okamoto

**Affiliations:** 1 Department of Nutritional Epidemiology and Shokuiku, National Institutes of Biomedical Innovation, Health and Nutrition, Tokyo, Japan; 2 Department of Nutrition and Food Science, Graduate School of Humanities and Sciences, Ochanomizu University, Tokyo, Japan; 3 Department of Human Nutrition, Seitoku University, Chiba, Japan; 4 Department of Nutrition, Faculty of Nutrition, Kobe Gakuin University, Hyogo, Japan; 5 Department of Clinical Dietetics and Human Nutrition, Faculty of Pharmaceutical Sciences, Josai University, Saitama, Japan; 6 Department of Health and Nutrition, Osaka Shoin Women's University, Osaka, Japan

**Keywords:** Women, Children, Mothers, Dietary intake, Japan, BDHQ, brief-type, self-administered diet history questionnaire

## Abstract

A growing body of evidence from Western countries shows that the presence of children in households is associated with the dietary intake of adults, but little is known about this relationship in non-Western countries with different food cultures. Our aim was to examine whether dietary intake was different with respect to the presence of young children in the home among Japanese married women. Subjects were Japanese married women (aged 23–44 years) living with children aged less than 5 years (*n* 73) and married women who did not have children (*n* 85). Data regarding habitual dietary intake were obtained using a validated, self-administered diet history questionnaire. A cross-sectional comparison between women with young children and women without children was conducted using ANCOVA adjusted for potential confounding factors. Women with young children had a significantly greater intake of protein, carbohydrates, Na, Zn and Cu than did women without children. Intake of cereals, pulses and sugar was significantly higher among mothers than among non-mothers. Intake of both alcoholic and non-alcoholic beverages was significantly higher among non-mothers than among mothers. Thus, the presence of young children at home might influence women's intake of macronutrients and some minerals, especially Na, and beverages among Japanese married women. Our findings suggest that effective dietary interventions among Japanese mothers with young children may differ from those of married women without children.

Dietary behaviour in the early stages of life could influence dietary intake later on in life^(^^1^^–^^3^^)^ and could even affect the future risk of non-communicable diseases^(^^4^^)^. Improving dietary habits of children is an important public health issue. Similarities in dietary intake among parents and their children have been observed in several studies^(^^5^^,^^6^^)^. A systematic review reported various similarities such as energy and macronutrient intake^(^^5^^)^. A more recent US study observed the relationships between diet quality and energy intake of parents and those of their children^(^^7^^)^. Thus, children's diet is regarded as being influenced primarily by that of their parents^(^^6^^)^.

On the other hand, adults’ lifestyle may also be affected by having children. The transition to parenthood can have a large impact on health and related behaviours such as increased BMI^(^^8^^,^^9^^)^ and decreased physical activity^(^^10^^–^^12^^)^. Several studies have compared dietary intake between parents and non-parents, and a significant difference in the intake of several nutrients and food items was observed^(^^12^^–^^16^^)^. For example, US mothers reported greater intake of sugar-sweetened beverages, total energy and saturated fat than non-mothers^(^^12^^)^. In another US study, the presence of children in the household was associated with significantly higher total and saturated fat intake among adults^(^^13^^)^. A UK study showed that women who had children under the age of 16 years had higher vegetable intake and lower fruit intake than did women without children under the age of 16 years^(^^14^^)^. Finnish women who had children were shown to have a dietary intake closer to dietary guidelines, such as greater consumption of fruits and vegetables^(^^15^^)^, than did non-mothers. Moreover, a previous study in the USA reported that the presence of a young child was positively associated with fruit and vegetable consumption^(^^16^^)^. Additionally, some longitudinal studies have examined the change in dietary intake during the transition to parenthood^(^^17^^–^^22^^)^. Among these studies, some reported that dietary patterns of women starting a family had changed unfavourably^(^^21^^,^^22^^)^. For instance, a Canadian study showed that first-time mothers decreased their fruit intake^(^^21^^)^, while an Australian study reported that women starting a new family increased their energy intake and consumption of high-fat foods, sugar, fruit and cooked vegetables^(^^22^^)^. There have been mixed results reported regarding the association between the presence of children in a household and the dietary intake of adults; however, despite a stronger association between dietary intake in children and their parents being observed in countries other than European countries and the USA^(^^5^^)^, we are unaware of any comparable research reported in Asian countries, including Japan, where the food culture is quite different from that of Western countries. Typically, the diet consumed by Japanese people is characterised by high intakes of rice, soyabean products, fish, seaweeds and green tea, as well as lower fat intake than that of Western countries^(^^23^^,^^24^^)^. To obtain reliable scientific evidence on the presence of children in a household and the dietary intake of adults, it will be necessary to accumulate studies of this kind conducted in various regions with different social and cultural backgrounds. Furthermore, there have been few reports on whether the presence of children influences overall nutrient intake, not only specific nutrients, in married women.

It has also been reported that mothers have stronger influences on their children's dietary intake than do fathers^(^^7^^,^^25^^–^^27^^)^, and maternal modelling of healthy food habits can influence children's diet quality^(^^28^^)^. Understanding the difference in dietary intake between women living with young children and those living without children will be important in proposing dietary strategies for the health of not only mothers, but also the next generation.

Here, we examined the influence of the presence of young children in the home on women's dietary intake. The objective of this study was to characterise the difference in dietary intake profiles of married women with children younger than 5 years old and married women without children, in Japan.

## Methods

### Procedure

This report describes a cross-sectional, self-administered questionnaire study (the POTATO study) conducted between June and December 2014 and between July and August 2015. Married women aged 23–44 years, with or without a preschool child or children younger than 5 years old, living in Japan, were recruited via an advertisement for volunteer collaborators (snowball sampling). A collaborator explained the survey's purpose and outline using a document distributed to a total of 353 married women who were asked to complete questionnaires about dietary habits and lifestyle. To compare the dietary intake of mothers of preschool children (younger than 5 years old) and women without children, inclusion criteria for subjects’ age was set between 23 and 44 years, because the birth rate was highest for women between the ages of 30 and 34 years and dropped at 40 years in Japan^(^^29^^)^. A total of 241 women completed both questionnaires (response rate = 68·3 %). This study was conducted according to the guidelines laid down in the Declaration of Helsinki, and all procedures involving human subjects were approved by the Ethics Committee of Seitoku University (no. H25U017). Written informed consent was obtained from all the participants.

### Study population

The present analyses were limited to those women who either lived with husbands only or with husbands and children aged younger than 5 years (*n* 232). We excluded the following respondents: those who were receiving nutritional counselling (*n* 2), those who were pregnant or lactating (*n* 63), those who reported energy intake less than half the energy requirement for the lowest physical-activity category according to the Japanese Dietary Reference Intakes 2015^(^^30^^)^, and those reporting equal to or more than 1·5 times the energy requirement for the highest physical activity category (*n* 3). There were no missing data on the variables analysed.

**Fig. 1.**
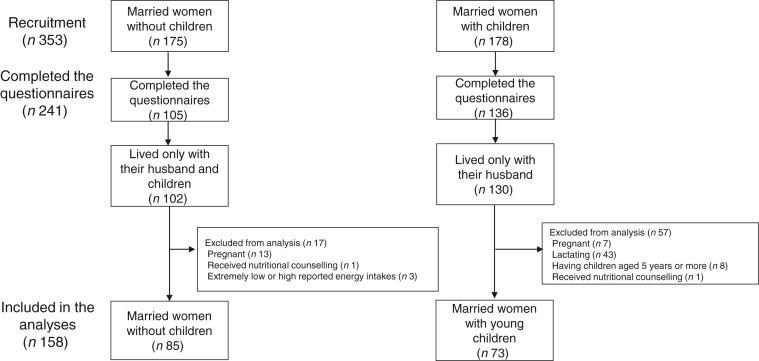
Participation in the POTATO study comparing the dietary intake of Japanese married women with respect to the presence of young children in the home.

According to the number of children reported in the lifestyle questionnaire, the subjects were categorised into two groups – women with children younger than 5 years old and women without children. Those who had children aged over 5 years were excluded (*n* 8). The final sample thus consisted of 158 Japanese married women aged 25–44 years categorised into the following two groups: subjects with young children (*n* 85) and without children (*n* 73) (Fig. 1).

### Dietary assessment

Dietary habits during the preceding month were assessed using a previously validated brief-type, self-administered diet history questionnaire (BDHQ)^(^^31^^,^^32^^)^. The BDHQ is a structured questionnaire that includes questions about intake frequencies of selected foods commonly consumed in Japan, general dietary behaviours and usual cooking methods. The daily intake estimates for foods (fifty-eight items in total), energy and selected nutrients were calculated using an *ad hoc* computer algorithm developed for the BDHQ, which was based on the Standard Tables of Food Composition in Japan^(^^33^^)^. Validity of the BDHQ and its structure, as well as the methods used to calculate dietary intake, have been detailed as referenced above. In a previous study of ninety-two women aged 31–69 years, the median Pearson's correlation coefficients of nutrient intake between the BDHQ and 16-d weighed dietary records was 0·54 (range 0·34–0·87), and the median Spearman's correlation coefficient of food intake was 0·44 (range 0·14–0·82)^(^^31^^,^^32^^)^. Although dietary supplement use was queried in the lifestyle questionnaire, intake via supplements was not included in the analysis because of the lack of a reliable composition table for dietary supplements in Japan.

### Assessment of lifestyle variables

In the lifestyle questionnaire, the subjects reported their educational background (junior high school, high school, junior college or vocational technical school, or university); working status (full-time, part-time, other); household income (less than 2 million yen/year, 2 million to 6 million yen/year, 6 million to 10 million yen/year, more than 10 million yen/year); current smoking status (yes or no); and dietary supplement use (yes or no). Data regarding self-reported body height (cm) and weight (kg) were also obtained from the BDHQ. BMI was calculated as body weight (kg) divided by the square of body height (m^2^). On the basis of their reported home address, each participant was grouped into one of six residential blocks (Hokkaido and Tohoku, Kanto, Hokuriku and Tokai, Kinki, Chugoku and Shikoku, or Kyushu), and into three categories according to population size (city with a population ≥1 million, city with a population <1 million, or town and village).

### Statistical analysis

All statistical analyses were performed using SAS statistical software, version 9.4 (SAS Institute Inc.). All reported *P* values were two-tailed, with *P*<0·05 considered statistically significant. Characteristics of the groups with and without children were compared using independent-samples *t* tests for continuous variables and the χ^2^ test for categorical variables.

All the selected nutrient and food intake values were energy-adjusted using the density method (i.e. the percentage of energy for energy-providing nutrients and their amounts per 1000 kcal (4184 kJ) for food groups and other nutrients) to minimise the influence of dietary misreporting, which has caused ongoing controversy in studies that collect dietary information via self-reported instruments.

We examined the difference in the selected nutrient and food intake between subjects with young children and without children by using ANOVA. Additionally, multivariate adjusted mean, with standard error, for nutrient and food intake was calculated for subjects with young children or without children. Potential confounding factors considered in the analysis were those indicating differences between the two groups (*P* < 0·1), namely, age (continuous variable), working status, annual household income, educational background and size of residential area, as detailed above. The categories of educational background and annual household income were regrouped due to the sample sizes of certain categories: *n* 0 for junior high school and *n* 1 for less than 2 000 000 yen/year. The adjusted nutrient and food intake differences between the two groups were assessed by ANCOVA. To perform the ANCOVA tests, the number of participants required to detect a medium effect size (*f* = 0·25) with a significance level equal to 0·05, statistical power of 0·8 and five covariates was estimated to be 128 in total, according to the power analysis using G*Power 3^(^^34^^)^.

## Results

Basic characteristics of subjects are shown in Table 1. The mean age of the subjects was 32·5 (sd 4·5) years. The mean number of participants’ children was 1·4 (sd 0·5). Compared with subjects with young children, those without children were younger, had a higher proportion of full-time workers, a lower proportion of annual household incomes less than 6 million yen per year, and a higher proportion of dwellers living in a city with a population ≥1 million. No significant differences were observed in BMI, energy intake, and the proportion of current smokers, supplement users, educational backgrounds, survey year, and residential blocks between women with and without children.

**Table 1.** Basic characteristics of 158 married women with or without young children at home (Mean values and standard deviations; numbers and percentages)

	Total (*n* 158)		With young children (*n* 73)		Without children (*n* 85)		P*
	*n*	%	*n*	%	*n*	%	
Number of children							–
Mean	–		1·4		–		
sd			0·5				
Number of children							
1	–		48	66·7	–		–
2	–		23	30·6	–		–
More than 3	–		2	2·8	–		–
Age (years)							<0·001
Mean	32·5		33·9		31·3		
sd	4·5		4·2		4·4		
BMI (kg/m^2^)							0·135
Mean	20·4		20·7		20·1		
sd	2·6		2·8		2·3		
Energy intake (kJ/d)							0·692
Mean	6903		6961		6853		
sd	1702		1707		1706		
Current smoking							0·519
Yes	6	3·8	2	2·7	4	4·7	
No	152	96·2	71	97·3	81	95·3	
Dietary supplement user							0·385
Yes	42	26·6	17	23·3	25	29·4	
No	116	73·4	56	76·7	60	70·6	
Educational background							0·081
University or higher	113	71·5	47	64·4	66	77·7	
Junior college or vocational technical school	35	22·2	22	30·1	13	15·3	
High school	10	6·3	4	5·5	6	7·1	
Working status							0·042
Full-time	73	46·2	26	35·6	47	55·3	
Part-time	29	18·4	15	20·6	14	16·5	
Others	56	35·4	32	43·8	24	28·2	
Annual household income							0·039
Less than 6 000 000 yen	70	44·3	40	54·8	30	35·3	
6 000 000 yen to 10 000 000 yen	65	41·1	23	31·5	42	49·4	
More than 10 000 000 yen	23	14·6	10	13·7	13	15·3	
Survey year							0·731
2014	117	74·1	55	75·3	62	72·9	
2015	41	26·0	18	24·7	23	27·1	
Residential block							0·343
Hokkaido and Tohoku	13	8·2	4	5·5	9	10·6	
Kanto	84	53·2	45	61·6	39	45·9	
Hokuriku and Tokai	15	9·5	6	8·2	9	10·6	
Kinki	18	11·4	8	11·0	10	11·8	
Chugoku and Shikoku	13	8·2	6	8·2	7	8·2	
Kyushu	15	9·5	4	5·5	11	12·9	
Size of residential area							0·027
City with a population ≥1 million	61	38·6	20	27·4	41	48·2	
City with a population <1 million	84	53·2	46	63·0	38	44·7	
Town or village	13	8·2	7	9·6	6	7·1	

* *P* values between married women with and without children. Means for continuous values were compared by using independent-samples *t* tests, and proportions for categorical values were compared using the χ^2^ test.

Table 2 shows the differences in nutrient intake between subjects with young children and without children. Women with young children had a significantly higher intake of Na than did women without children in the crude model. Even after adjusting for potential confounding factors, the intake of Na was significantly different between the two groups. Additionally, in the multivariate models, women with young children also had a higher intake of proteins, carbohydrates, Zn and Cu than did those without children.

**Table 2.** Daily nutrient intake among 158 married women with and without young children at home (Mean values with their standard errors)

	Crude model						Multivariate model†					
	With young children (*n* 73)		Without children (*n* 85)				With young children (*n* 73)		Without children (*n* 85)			
Nutrient	Mean	se	Mean	se	Difference	*P*‡	Mean	se	Mean	se	Difference	*P*§
Protein (% energy)	15·6	0·3	14·9	0·3	0·7	0·067	15·8	0·5	15·0	0·4	0·8	0·046*
Fat (% energy)	28·0	0·5	28·5	0·5	0·5	0·481	27·3	0·9	28·1	0·8	0·8	0·291
SFA (% energy)	7·8	0·2	7·9	0·2	0·1	0·724	7·5	0·3	7·7	0·3	0·2	0·663
Carbohydrate (% energy)	53·4	0·7	51·8	0·7	1·6	0·097	53·9	1·2	51·2	1·1	2·7	0·012*
Total dietary fibre (g/4184 kJ)	7·2	0·2	6·8	0·2	0·4	0·194	7·0	0·4	6·5	0·3	0·5	0·145
Vitamin A (μg RAE/4184 kJ)||	459	27	445	20	14	0·673	412	40	412	37	0	0·999
Vitamin B_1_ (mg/4184 kJ)	0·45	0·01	0·44	0·01	0·01	0·454	0·44	0·02	0·42	0·01	0·02	0·303
Vitamin B_2_ (mg/4184 kJ)	0·71	0·02	0·73	0·02	0·02	0·602	0·70	0·03	0·71	0·03	0·01	0·517
Niacin (mg NE/4184 kJ)¶	9·4	0·3	9·3	0·3	0·1	0·792	9·3	0·5	9·3	0·5	0·0	0·945
Vitamin B_6_ (mg/4184 kJ)	0·72	0·02	0·71	0·02	0·01	0·718	0·71	0·03	0·69	0·03	0·02	0·655
Vitamin B_12_ (μg/4184 kJ)	5·01	0·26	4·40	0·27	0·61	0·109	5·54	0·46	4·85	0·42	0·69	0·093
Folate (μg/4184 kJ)	192	8	197	7	5	0·609	188	14	192	13	4	0·729
Vitamin C (mg/4184 kJ)	61	3	62	3	1	0·650	61	5	60	4	1	0·839
Na (salt-equivalent) (g/4184 kJ)††	5·8	0·1	5·3	0·1	0·5	0·004*	6·4	0·2	5·8	0·2	0·6	0·001*
K (mg/4184 kJ)	1422	40	1408	37	14	0·801	1370	67	1350	63	20	0·739
Ca (mg/4184 kJ)	305	10	290	10	15	0·302	294	17	280	16	14	0·360
Mg (mg/4184 kJ)	139	3	135	3	4	0·302	139	5	135	5	4	0·325
Fe (mg/4184 k)	4·45	0·12	4·34	0·11	0·11	0·506	4·54	0·20	4·39	0·19	0·15	0·404
Zn (mg/4184 kJ)	4·60	0·06	4·47	0·06	0·13	0·158	4·56	0·11	4·37	0·10	0·19	0·048*
Cu (mg/4184 kJ)	0·64	0·01	0·61	0·01	0·03	0·057	0·65	0·02	0·61	0·02	0·04	0·009*

NE, niacin equivalents; RAE, retinol activity equivalents.

* *P* < 0·05.

† The model included age (continuous variable), working status (full-time, part-time, or other), annual household income (less than 6 000 000 yen/year, 6 000 000 to 10 000 000 yen/year, or more than 10 000 000 yen/year), educational background (high school, junior college or vocational technical school, or university), and size of residential area (city with a population ≥1 million, city with a population <1 million, or town/village).

‡ *P* values between married women with and without children (ANOVA).

§ *P* values between married women with and without children (ANCOVA).

|| Sum of retinol, β-carotene/12, α-carotene/24 and cryptoxanthin/24.

¶ Sum of niacin and protein/6000.

†† Na × 2·54.

Intake of pulses and sugar was higher among women with young children than women without them, while intake of oil, alcoholic beverages, non-alcoholic beverages, and fruit and vegetable juices was higher among women without children than women with them (Table 3). In multivariate-adjusted models, similar results were observed with the addition of cereal intake.

**Table 3.** Daily food intake among 158 married women with and without young children in the home (Mean values with their standard errors)

	Crude model						Multivariate model†					
	With young children (*n* 73)		Without children (*n* 85)				With young children (*n* 73)		Without children (*n* 85)			
Food intake (g/4184 kJ)	Mean	se	Mean	se	Difference	*P*‡	Mean	se	Mean	se	Difference	*P*§
Cereals	219·4	6·7	206·9	6·5	12·5	0·186	222·5	11·6	201·6	10·8	20·9	0·046*
Rice	156·6	7·0	151·7	7·0	4·9	0·618	152·7	11·9	137·2	11·1	15·5	0·150
Noodles	39·5	3·3	34·8	2·6	4·7	0·261	50·1	4·9	44·0	4·6	6·1	0·170
Bread	23·3	1·7	20·4	2·0	2·9	0·296	19·8	3·3	20·4	3·1	0·6	0·844
Pulses	126·8	7·3	99·2	5·1	27·6	0·002*	145·7	10·7	111·3	10·0	34·4	0·001*
Potatoes	25·6	1·7	23·0	2·2	2·6	0·349	25·3	3·4	20·7	3·2	4·6	0·136
Sugar and confectioneries	44·2	3·7	45·6	2·6	1·4	0·739	49·4	5·4	50·1	5·1	0·7	0·885
Sugar	3·0	0·2	2·3	0·2	0·7	0·024*	3·0	0·4	2·3	0·4	0·7	0·042*
Confectioneries	41·2	3·7	43·4	2·6	2·2	0·623	46·4	5·5	47·8	5·1	1·4	0·774
Oil	9·5	0·4	10·8	0·4	1·3	0·013*	8·7	0·7	10·4	0·6	1·7	0·005*
Fruits	33·7	3·0	29·2	2·5	4·5	0·245	32·0	4·6	27·1	4·3	4·9	0·240
Total vegetables	166·9	10·1	163·9	8·3	3·0	0·819	157·9	16·2	152·4	15·0	5·5	0·707
Green and yellow vegetables	64·7	4·7	61·5	4·2	3·2	0·612	56·8	7·7	54·0	7·2	2·8	0·683
Other vegetables	81·3	5·6	81·9	4·3	0·6	0·933	79·2	8·6	77·5	8·0	1·7	0·835
Pickled vegetables	5·5	0·8	4·7	0·7	0·8	0·435	7·8	1·3	6·4	1·2	1·4	0·234
Mushrooms	8·4	0·6	9·0	0·7	0·6	0·499	7·5	1·1	8·1	1·1	0·6	0·598
Seaweeds	7·0	0·7	6·8	0·7	0·2	0·854	6·6	1·1	6·5	1·1	0·1	0·910
Alcoholic beverages	37·4	10·4	73·6	13·1	36·2	0·036*	34·6	19·9	89·6	18·5	55·0	0·003*
Non-alcoholic beverages	254·8	19·8	319·0	21·6	64·2	0·032*	280·4	37·0	347·5	34·4	67·1	0·045*
Fruit and vegetable juice	17·1	3·0	31·3	5·0	14·2	0·020*	21·8	7·5	36·5	6·9	14·7	0·030*
Green, black and oolong tea	133·2	19·1	179·9	16·5	46·7	0·064	165·4	31·4	204·5	29·3	39·1	0·168
Coffee	81·8	10·3	82·9	10·9	1·1	0·941	59·6	18·3	73·6	17·1	14·0	0·397
Soft drinks	22·7	3·5	24·8	4·6	2·1	0·721	33·6	7·3	33·0	6·8	0·6	0·924
Fish and shellfish	40·9	2·7	36·1	3·0	4·8	0·246	48·0	4·9	40·6	4·6	7·4	0·099
Meats	43·9	1·9	45·7	1·6	1·8	0·457	40·0	3·0	42·0	2·8	2·0	0·465
Eggs	20·8	1·4	21·1	1·4	0·3	0·898	21·9	2·4	22·7	2·2	0·8	0·705
Dairy products	64·1	6·4	63·5	5·2	0·6	0·940	42·2	10·0	46·2	9·3	4·0	0·662

* *P* < 0·05.

† The model included age (continuous variable), working status (full-time, part-time, or other), annual household income (less than 6 000 000 yen/year, 6 000 000 to 10 000 000 yen/year, or more than 10 000 000 yen/year), educational background (high school, junior college or vocational technical school, or university), and size of residential area (city with a population ≥1 million, city with a population <1 million, or town/village).

‡ *P* values between married women with and without children (ANOVA).

§ *P* values between married women with and without children (ANCOVA).

## Discussion

To the best of our knowledge, the present study is the first to examine the difference in nutrient and food intake between Japanese married women with young children in the household and those without children. We found that there were several differences in nutrient and food intake profiles between the two groups of subjects. Parenthood is regarded as not only a period of transition in a person's life, but also a period of change in dietary habits. Dietary intake has been compared between parents and non-parents in overseas studies, many of which reported differences in fat intake. For example, in the USA, mothers with children aged 5 years old or younger had a greater intake of total energy and percentage of SFA than did women without children^(^^12^^)^. Another study also reported that the presence of children younger than 17 years old in the household was associated with greater intake of total fat and saturated fat^(^^13^^)^. Moreover, in a longitudinal study in the USA, adults who had new children in their homes had a greater intake of saturated fat than did non-parents^(^^19^^)^. Similarly, a Canadian study observed that the total intake of fat increased among new parents while it decreased among non-parents^(^^21^^)^. In contrast, our results showed that intake of other energy-providing nutrients, namely protein and carbohydrates, was higher among women with young children than among non-parents, while we observed no significant difference in the intake of fat and SFA between women with young children and those without them. These differences in nutrient intake reflected greater intake of cereals, pulses and sugars among subjects with young children in the present analysis. It has been reported that intake of fat and SFA is smaller in the Japanese population than in Western populations^(^^24^^)^; moreover, this might have led to the differences between the present study's findings and those studies conducted in Western countries. Although we could not obtain children's dietary intake in cereals and pulses, a higher intake of these might reflect the children's dietary intake. Parents’ food selections might be influenced by the presence of preschool children^(^^35^^)^. Asakura *et al.*^(^^36^^)^ reported that, according to dietary records of children aged 3–6 years, well-milled rice, soya sauce, egg, carrot, onion, milk, potato, tofu and pork are frequently consumed foods, and these results are partially consistent with our findings. On the other hand, a difference in cereal intake was observed only in the multivariate-adjusted model, where age was significantly associated with lower cereal intake. This could be explained by the older age of women in the group with children than that of women in the group without children. This trend of higher grain intake among younger women is consistent with previous results in Japan^(^^23^^)^, although this might not be the influence of the presence of children in the households. To determine whether children's dietary intake or preferences influence the dietary habit of their parents in the Japanese population, further studies are needed.

In the present study, mothers had higher intakes of Na, Zn and Cu than did non-parents. One Japanese study reported that white rice was the largest contributor to the intake of Zn and Cu, accounting for approximately 20 % of each^(^^37^^)^. This study also showed that pulses and nuts contributed to the intake of these nutrients, which might explain why our subjects with young children had higher intakes of Zn and Cu than did the non-parents. The estimated difference in Na intake between the two groups could be as much as 1 g/d. One study reported that more than 60 % of Japanese women's Na intake comes from seasonings such as soya sauce and salt^(^^38^^)^. However, we could not compare the intake of seasonings, because validity of the intake of these food items has not been examined in the BDHQ; thus the amounts of seasonings consumed could have been different between mothers and non-mothers. Although we could not refer to the effects of transition to parenthood in this cross-sectional study, mothers may need to be more conscious of their Na intake than they were when they did not have children. Meanwhile, in Eastern Asia, particularly Japan, Na intake is greater than that in other countries^(^^39^^)^, which is a major health problem^(^^40^^)^. Our findings, therefore, may illustrate the need for implementing an effective strategy for reducing Na intake in the target population. Furthermore, children's dietary habits could be influenced by the dietary habits of their parents^(^^6^^)^. Therefore, health-promotion programmes, including nutrition counselling, may need to draw mothers’ attention to Na intake, not only for themselves, but also for their children, who could carry these dietary habits into the future.

Our subjects with young children had a lower intake of alcoholic beverages. This result is consistent with a US study showing that motherhood is associated with marked reductions in alcohol consumption^(^^41^^)^. Alcohol reduction during pregnancy and lactation could continue after these periods. Since alcohol intake, in turn, affects intake of foods and nutrients, the differences in dietary intake observed in the present study could have been influenced by the intake of alcoholic beverages. For example, one study showed that moderate or heavy drinkers had a lower intake of carbohydrates than did non-drinkers among Finnish women^(^^42^^)^. Moreover, one study in the USA comparing dietary intake on a drinking day with that of a non-drinking day observed lower intakes of discretionary oil and solid fat on a non-drinking day among women^(^^43^^)^, which is partially consistent with our findings. Dietary intake of married women could be affected not only by the presence of young children at home, but also by behavioural changes during pregnancy and lactation. Interestingly, we found that mothers also had a lower intake of non-alcoholic beverages, particularly fruit and vegetable juices, than did non-mothers. Another US study showed that mothers with young children had a greater intake of sugar-sweetened beverages than did women without children, which differs from the results of our findings^(^^12^^)^. Among Japanese young women, it has been reported that a higher intake of soft drinks was associated with lower intakes of protein, dietary fibre, vitamins and minerals, even though their intake of soft drinks was lower than that of the US population^(^^44^^)^. With a few exceptions, this result is consistent with our results. Additionally, the present study showed that there was no difference in reported energy intake between the two groups. A Canadian study on beverage consumption patterns reported that a smaller amount of beverage intake was associated with lower energy intake^(^^45^^)^. It could be said, therefore, that Japanese mothers derive more energy or nutrients from foods, than from beverages. Further studies are needed to examine beverage consumption patterns and their effect on dietary intake in the Japanese population, in addition to the influence of the presence of young children in a home on beverage consumption. Nevertheless, our findings indicated that beverage intake habits could change during the transition to motherhood.

Our study has several limitations. First, the subjects of this study were recruited based on a snowball-recruitment strategy and were, therefore, not a random sample of Japanese adult women. The advertisement was distributed mainly via graduates of several universities; thus, all the subjects might have had relatively high social and economic status. Compared with dietary intake data of women aged 30–39 years in the National Health and Nutrition Survey in Japan, 2016^(^^23^^)^, which represented the general Japanese population, the fat and carbohydrate energy ratio was slightly lower in the current participants. Due to our recruitment strategy, women who were interested in nutrition were more likely to participate, thus selection bias could not be ruled out. This might explain the small differences between the groups. Moreover, data were only collected among women who lived with their husbands, either with or without children younger than 5 years of age. This strategy was employed to examine the effect made by the presence of children; however, results cannot be extrapolated beyond that. Second, the period of recruitment and the consequent data collection differed based on survey years (2014: June to December; 2015: July to August); thus, the difference in dietary intake by seasons may need to be considered. Because the number of subjects participating in 2014 and 2015 did not differ between the groups, we did not consider this factor in the multivariate analyses. Although the number of subjects was not sufficiently large enough to make a comparison by survey seasons, the results did not differ when we adjusted for the survey years in addition to the multivariate adjustment models (data not shown). Additionally, validation of the BDHQ used 4-d dietary records conducted in each of the four seasons over the period of 1 year as the reference method for minimising the effect of seasonal dietary intake variations^(^^32^^)^. Furthermore, the aforementioned study examined the validity by using not only a single season's results of the questionnaire but also the mean values of four seasons’ results; thus, we considered that the season variation could be minimised. Third, we were unable to obtain nutrient intake information regarding dietary supplementation. Approximately one-quarter of each group used dietary supplements; thus, the intake from dietary supplements could have affected the results to some extent. Moreover, supplement use could not be evaluated because of the lack of a reliable composition table of dietary supplements in Japan. However, the proportion of dietary supplement users did not differ between the two groups, which may support the robustness of the present findings. Fourth, there could be other confounding factors in addition to those we examined. For example, among women having young children, some subjects may have been on child-care leave from their full- or part-time jobs. In this case, the working status was considered as ‘other’; therefore, the effect on the results from such factors could not be ruled out. Finally, this is a cross-sectional study, and therefore causal relationships were not clarified. It is possible that the women with children already had concrete dietary behaviours before they transitioned to motherhood. Without a longitudinal study, therefore, we cannot say definitively that the presence of a child is responsible for a positive/negative change in dietary habits.

### Conclusions

This cross-sectional study showed that there were several differences in the intake of nutrients and foods between married Japanese women with young children and those without children. Our findings suggest that the presence of young children might influence a mother's intake of macronutrients and some minerals, as well as beverage intake habits. Moreover, our findings indicate that mothers especially need to attend to their Na intake. Thus, health-promotion programmes or nutrition counselling might need to pay greater attention to this special period in a mother's life in which great changes can occur in her dietary habits. Further studies, including longitudinal studies, are needed to clarify the association between the presence of children in households and adults’ dietary habits.
